# Smartphone intervention to optimize medication assisted treatment outcomes for opioid use disorder: study protocol for a randomized controlled trial

**DOI:** 10.21203/rs.3.rs-2511936/v1

**Published:** 2023-02-15

**Authors:** Ron G Thompson, Mary Bollinger, Michael Mancino, Deborah Hasin, Xiaotong Han, Keith A Bush, Clint D Kilts, G Andrew James

**Affiliations:** 1 Department of Psychiatry, University of Arkansas for Medical Sciences, Little Rock, AR; 2 Department of Epidemiology, Columbia University, New York City, NY

**Keywords:** Opioid-Related Disorders (D009293), Mobile Applications (D063731), Opiate Substitution Treatment (D058850), Smartphone (D000068997), Behavioural Therapy (D001521)

## Abstract

**Background:**

Opioids accounted for 75% of drug overdoses in the United States in 2020, with rural states particularly impacted by the opioid crisis. While medication assisted treatment (MAT) with Suboxone remains one of the more efficacious treatments for opioid use disorder (OUD), approximately 40% of people receiving Suboxone for outpatient MAT for OUD (MOUD) relapse within the first 6 months of treatment. We developed the smartphone app-based intervention OptiMAT as an adjunctive intervention to improve MOUD outcomes. The aims of this study are to (1) evaluate the efficacy of adjunctive OptiMAT use in reducing opioid misuse among people receiving MOUD; and (2) evaluate the role of specific OpitMAT features in reducing opioid misuse, including the use of GPS-driven just-in-time intervention.

**Methods:**

We will conduct a two-arm, single-blind, randomized controlled trial of adults receiving outpatient MOUD in the greater Little Rock AR area. Participants are English-speaking adults ages 18 or older recently enrolled in outpatient MOUD at one of our participating study clinics. Participants will be allocated via 1:1 randomized block design to (1) MOUD with adjunctive use of OptiMAT (MOUD+OptiMAT) or (2) MOUD without OptiMAT (MOUD-only). Our blinded research statistician will evaluate differences between the two groups in opioid misuse (as determined by quantitative urinalysis conducted by clinical lab staff blinded to group membership) during the 6-months following study enrolment. Secondary analyses will evaluate if OptiMAT-usage patterns within the MOUD+OptiMAT group predict opioid misuse or continued abstinence.

**Discussion:**

This study will test if adjunctive use of OptiMAT improve MOUD outcomes. Study findings could lead to expansion of OptiMAT into rural clinical settings, and the identification of OptiMAT features which best predict positive clinical outcome could lead to refinement of this and similar smartphone app-based interventions.

**Trial registration:**

ClinicalTrials.gov identifier: NCT05336188, registered March 21, 2022, https://clinicaltrials.gov/ct2/show/NCT05336188.

## Administrative information

Note: the numbers in curly brackets in this protocol refer to SPIRIT checklist item numbers. The order of the items has been modified to group similar items (see http://www.equator-network.org/reporting-guidelines/spirit-2013-statement-defining-standard-protocol-items-for-clinical-trials/).

**Table:** 

Title {1}	**Smartphone intervention to optimize medication assisted treatment outcomes for opioid use disorder: study protocol for a randomized controlled trial**
Trial registration {2a and 2b}.	ClinicalTrials.gov identifier: NCT05336188, registered March 21, 2022, https://clinicaltrials.gov/ct2/show/NCT05336188
Protocol version {3}	OptiMat Protocol v4, July 11, 2022
Funding {4}	NIDA / NIGMS grant R01DA048022
Author details {5a}	**Ron G Thompson Jr** ^**1**^ **, Mary Bollinger** ^**1**^ **, Michael Mancino** ^**1**^ **, Deborah Hasin** ^**2**^ **, Xiaotong Han** ^**1**^ **, Keith A Bush** ^**1**^ **, Clint D Kilts** ^**1**^ **, G Andrew James** ^**1**^
	^1^Department of Psychiatry, University of Arkansas for Medical Sciences, Little Rock, AR ^2^Department of Epidemiology, Columbia University, New York City, NY
Name and contact information for the trial sponsor {5b}	University of Arkansas for Medical Sciences, Office of Regulatory Affairs, 4301 W. Markham Street, Little Rock, AR, 72205, 501-526-6876
Role of sponsor {5c}	Study sponsors and funders have no role in: study design; collection, management, analysis, and interpretation of data; writing of the report; and the decision to submit the report for publication. Study sponsors and funders have no authority over any of these decisions.

## Introduction

### Background and rationale {6a}

The United States is experiencing an opioid use crisis. Opioids were involved in 68,630 overdose deaths (over 188 people daily) in 2020, accounting for 75% of all US drug overdose deaths.(1) Rural states are particularly impacted by this growing crisis, as greater opioid dispensing rates and less accessibility to healthcare resources have resulted in opioid-related mortality rates up to four times greater than urban areas. As a rural state, Arkansas is no exception, having the second highest opioid dispensing rate in the nation (75.8 opioid prescriptions per 100 residents as compared to national average of 43.3 in 2020).(2)

The most efficacious therapy for opioid use disorder (OUD) is medication for OUD (MOUD) which uses opioid substitution (primarily methadone or buprenorphine) to alleviate craving and withdrawal symptoms to promote abstinence. Clinical trials have reported opioid abstinence rates as high as 75% during short-term inpatient MAT(3). However, an estimated 60% of OUD patients relapse within one year of initiating outpatient MAT therapy.(4) Adjunctive medication therapies during MAT (such as extended-release naltrexone) help attenuate relapse rates (reducing rates to ~40%)(4,5), but these adjunctive therapies likewise suffer from limited availability in rural areas.

Telemedicine has emerged as a potential solution to the geographic barriers limiting healthcare access in rural areas. Specifically, smartphone applications (“apps”) have been developed which administer brief motivational interventions to aid weight loss,(6–9) smoking cessation,(10–12) and alcohol use reduction.(13–15) These app-based interventions combine *self-monitoring* of caloric intake, exercise, or drug use with *personalized feedback* to shape users’ behavior. However, this technology has not been applied to OUD in rural states, where rates of OUD are increased relative to population size and MOUD access is limited by geographic and financial barriers.

Therefore, we developed OptiMAT (“Optimizing Medication Assisted Treatment”), a novel app-based intervention to reduce opioid relapse during outpatient MOUD. OptiMAT provides: self-monitoring of daily opioid use, opioid craving, and mood; personalized feedback on MOUD goal attainment; charts depicting pattern of self-assessment responses over time; health promotion information, including identifying nearby abstinence-supporting resources; daily reminders to complete logging activities; and a GPS-driven just-in-time intervention. Since OptiMAT is an adjunctive intervention, we will compare outcomes of patients receiving outpatient MOUD with OptiMAT (MOUD+OptiMAT) against outcomes of patients receiving only outpatient MOUD (MOUD-only).

### Objectives {7}

The primary objective of this study is to evaluate whether a smartphone app-based adjunctive intervention (OptiMAT) can reduce opioid misuse among patients receiving MOUD. The secondary objectives are to evaluate the role of specific OptiMAT app features in reducing opioid misuse, including its use of GPS-driven just-in-time intervention.

### Trial design {8}

We will recruit patients with opioid use disorder who have initiated outpatient MOUD to participate in a two-arm stratified randomized control trial (RCT). Participants will be randomized 1:1 within each stratum (comorbid chronic pain and comorbid use of alcohol and/or marijuana, as detailed below) to either the MOUD-only arm (treatment as usual) or the MOUD+OptiMAT arm (adjunctive treatment intervention).

Of note, participants from each study arm will also be invited to participate in a longitudinal functional neuroimaging substudy investigating neural mechanisms associated with recovery from opioid use disorder, to be conducted at the University of Arkansas for Medical Sciences (UAMS) Brain Imaging Research Center (BIRC). This neuroimaging substudy is not directly pertinent to the RCT methods and will be detailed in future publications.

## Methods: Participants, interventions and outcomes

### Study setting {9}

Participants will be recruited from outpatient MOUD clinics in the central Arkansas area. The primary study setting is the UAMS Psychiatric Research Institute (PRI). The primary recruitment site is the UAMS PRI Center for Addiction Services and Treatment (CAST) outpatient MOUD clinic. Secondary recruitment sites will be identified through treatment programs participating in the MATRIARC program (Medication Assisted Treatment Recovery Initiative for Arkansas Rural Communities), a partnership between UAMS and the Arkansas Department of Human Services designed to expand evidence-based treatment for opioid use disorders.

### Eligibility criteria {10}

Inclusion criteria for the trial are males and females, 18 years of age or older, who have initiated outpatient MOUD at CAST or an affiliated MAT clinic within the past three weeks. The patient must have completed the initial Intake session and at least one of the weekly individual therapy sessions. The RCT study has no exclusionary criteria. Note that past MOUD treatment, including initiating MOUD in a residential setting prior to initiating outpatient MOUD, is not exclusionary.

### Who will take informed consent? {26a}

Research coordinators will obtain written informed consent from potential trial participants at the UAMS CAST clinic or UAMS MATRIARC-affiliated clinic. We have obtained a partial waiver of HIPAA authorization so that clinical staff may identify patients meeting eligibility criteria and introduce those individuals to a research coordinator embedded within CAST or UAMS MATRIARC programs. The research coordinator will provide patients with IRB-approved advertisements describing the study. The research coordinator will also schedule an intake visit for interested patients, where patients will undergo the informed consent process (including full HIPAA authorization) in a private and confidential setting. We have also obtained a partial HIPAA waiver so that participants who need more time to decide may provide us with contact information (i.e., name and phone number) so that we may contact them at a later date.

### Additional consent provisions for collection and use of participant data and biological specimens {26b}

This trial will not collect biological specimens. Biological specimens collected for clinical use (i.e. urinalysis) will not be stored or used for research. Consistent with Open Science principles, data collected from this trial will be de-identified and shared with neuroimaging data repositories. The informed consent form clarifies this data sharing process with the participant, and that the shared de-identified data may be used for other purposes (e.g. methods development) beyond the scope of this trial.

## Interventions

### Explanation for the choice of comparators {6b}

Since OptiMAT is an adjunctive intervention, we will compare opioid misuse outcomes of patients receiving outpatient MOUD with OptiMAT (MOUD+OptiMAT) against outcomes of patients receiving only outpatient MOUD (MOUD-only).

### Intervention description {11a}

#### Outpatient MOUD procedures.

At their first clinical visit, patients provide a complete medical, substance use, and psychiatric history to their provider. Patients also provide a urine sample for drug testing and meet briefly with their provider for a psychiatric evaluation. Patients will then receive Suboxone (buprenorphine plus extended-release naltrexone) in 4mg doses until withdrawal symptoms are suppressed. Patients will attend a weekly individual session with their provider, and (pending COVID regulations) a weekly group session. Patients receive additional Suboxone prescriptions at their weekly individual sessions.

Clinical sites use a phased treatment approach, in which patients have different levels of monitoring at different phases of treatment. New patients begin treatment at Phase I (weekly on-site Suboxone administration with one week of take-home medication) and matriculate toward Phase IV (monthly on-site administration with one month of take-home medication) based on evidence of continued abstinence from opioid misuse. As detailed below, we will recruit new Phase I patients who have completed their intake therapy session and at least one weekly individual therapy session, thus conveying genuine intent for treatment.

#### Control condition: MOUD-only.

Participants randomized into the MOUD-only study arm will undergo treatment as usual (TAU) with Suboxone MOUD without adjunctive use of the OptiMAT smartphone app, as described above.

#### Experimental condition: MOUD+OptiMAT.

Participants randomized into the MOUD+OptiMAT study arm will continue with MOUD treatment with adjunctive use of the OptiMAT smartphone app. Adapted from HealthCall-S,(16) OptiMAT is a complementary relapse minimization intervention for MOUD providing (1) *self-monitoring* to promote awareness of opioid misuse, and (2) *personalized feedback* for motivating abstinence goals. Pilot studies have shown HealthCall-S’s efficacy in reducing alcohol use with or without comorbid use of other drug of abuse.(16–20) Our choice of the HealthCall-S app as the adapted technology is further motivated by our preliminary data demonstrating its efficacy in reducing alcohol use and risky sexual behavior among homeless young adults.(21)

##### Base Features.

Like its predecessors, OptiMAT consists of three features accessible to participants: (1) Self-monitoring (aka “Tracker”), (2) Personalized feedback (including “Graphs”), and (3) accessible Resources. Geographical Ecological Momentary Assessment (GEMA) will not be directly accessible; this feature will run in OptiMAT’s background to record participants’ geospatial locations and intervene when participants enter individually defined high lapse-risk environments.

###### (1) Self-monitoring and (2) Personalized feedback.

These features function interactively as brief interventions for reducing opioid use. Participants will be asked to complete daily assessments of (a) opioid use, (b) opioid craving, (c) withdrawal symptoms, (d) mood (stress, anger, and sadness), and (e) use of alcohol or marijuana. Participants will be asked to complete the assessments at their convenience and will receive daily reminders at a time they prefer. Self-ratings will consist of yes/no questions (for opioid, alcohol, and marijuana use) and number responses entered by either a number pad (for amount spent on opioids, number of beer equivalents, or number of times used marijuana) or a labeled sliding bar Likert scale (for craving, withdrawal, and mood scales). Participants will receive personalized feedback consisting of positive reinforcement for meeting abstinence goals, or encouragement and tips to change behavior if lapsed to opioid use and/or experienced high opioid craving. Rating sessions will conclude with graphs summarizing responses, additional encouragement and restating of goal reminders, and further guidance from the Tip Bank. These response graphs will also be available to participants through the Graphs toolbox.

###### (3) Abstinence-promoting Resources.

OptiMAT will provide personally targeted information to help participants maintain abstinence from opioid misuse, including (a) information to help manage withdrawal symptoms, such as encouragement that the symptoms will pass, (b) a Tip Bank with tips to reduce craving (such as distracting activities or guided relaxation), (c) contact information for local emergency resources. Since our OnTrack study suggested participants will be unlikely to explore the Tip Bank on their own, the Tip Bank will provide additional lapse prevention guidance as part of the personalized feedback.

UAMS has recently initiated “AR ConnectNow” a state-wide hotline for non-emergency crisis services including on-call counselors capable of providing treatment referrals for addiction. OptiMAT would repurpose AR ConnectNow resources through its Resources section as well as GEMA intervention, described below. Additional resources include 9-1-1 and up to 3 supportive contacts of the participant’s choice (their “support network”).

###### (4) Geographic ecological momentary assessment (GEMA).

Each OptiMAT participant will provide up to three physical locations where they report greatest risk for opioid use relapse. The research coordinator will program each location as a “hotspot” using latitude and longitude coordinates. For privacy purposes, addresses will be identified as the nearest street intersection rather than specific people or places. OptiMAT will create a “geofence” around each hotspot location. The default geofence is a 1/16^th^ mile radius (~300 feet) around the hot spot, but this is adjustable based on urbanicity, rurality, or participant’s privacy concerns. Entering the geofenced perimeter triggers a push notification urging the participant to leave the hotspot. Remaining in the hot spot for 5 minutes triggers a second push notification asking the participant to report their craving intensity on the sliding bar Likert scale. Remaining in the hot spot for an additional 5 minutes (10 minutes total) prompts the participant to call a contact from their support network.

###### (5) Data security and confidentiality.

Given the sensitive nature of self-reported smartphone data for this project, OptiMAT employs the following security features to ensure participant confidentiality. First, all data will be stored on a HIPAA-compliant, HITRUST certified secure database managed by Enqbator. Second, data collected by OptiMAT will only be stored temporarily on the phone until the phone detects a secure network (a trusted Wi-Fi or satellite network), at which point data will be uploaded to the above server and erased from the phone. Finally, participants will be required to enable passcode protected screen lock on their phones to prevent unauthorized access.

### Criteria for discontinuing or modifying allocated interventions {11b}

Participation in this study is voluntary, and participants may withdraw from this study at any time. Study investigators will not collect any new data from participants who withdraw, but will retain any data already collected. Participant who withdraw from the study will not be allowed back into the study at a later date.

The principal investigator (GAJ) may withdraw participants from the study if 1) they do not follow study instructions, 2) they provide misleading or false information during study assessments, or 3) the principal investigator determines it is not in their best interest to continue. Note that we are documenting daily app usage and missed urinalysis test, so participants not using the app or missing clinical appointments are not valid reasons for withdrawing them from this study.

### Strategies to improve adherence to interventions {11c}

OptiMAT will send daily reminders for participants to log their daily self-monitoring assessment. Additionally, study staff will review mean OptiMAT use (percent days per month) at each of the participants’ monthly follow-up visits and provide strategies to maintain consistent OptiMAT use.

### Relevant concomitant care permitted or prohibited during the trial {11d}

No concomitant care is prohibited during the trial.

### Provisions for post-trial care {30}

Participant outcomes will be monitored for 6 months post study enrollment. However, participants in the MOUD+OptiMAT arm will be permitted to continue using OptiMAT until study closure. This study offers no compensation to those who suffer harm from trial participation.

### Outcomes {12}

#### Primary outcome.

The RCT has one primary outcome variable: percentage of clinically acquired urinalysis tests positive for opioid misuse. Urinalysis drug testing will consist of solid-phase enzyme immunoassay with positive tests analyzed by LC-MS for metabolite quantification. Urinalysis will assess opioid misuse, defined as presence of opioids in the urine other than Suboxone and its metabolites, including: methadone, fentanyl (11 metabolites), oxycodone, phencyclidine, propoxyphene, and buprenorphine or norbuprenorphine, as well as other drugs of abuse including amphetamines, barbiturates, ethanol, benzodiazepines, THC, and cocaine (benzoyecgonine). Urinalysis drug tests will be acquired at each clinical visit, which occur weekly when participants initiate treatment but may occur less frequently (e.g. every two weeks) as participants maintain sobriety. We will calculate percent positive urinalysis tests out of total possible urinalysis tests to generate a Treatment Effectiveness Score (TES) describing participant outcome relative to other participants in the RCT.(22) Note that TES treats missed urinalysis tests and positive urinalysis results equivalently, thus accounting for treatment cessation. TES also accounts for the frequency of urinalysis tests, which varies as patients matriculate from Phase I (weekly) to Phases II-IV (biweekly to monthly) as patients demonstrate continued abstinence from opioid misuse.

#### Secondary outcome.

The RCT will have several secondary outcome variables: self-reported days of opioid misuse; time to opioid lapse; and time to treatment discontinuation. Self-reported days of opioid misuse will be assessed using the TimeLine Follow-Back Calendar (TLFB).(23) The TLFB is a calendar that provides cues (such as holidays) to prompt participants to recall days of opioid misuse over the past month. TLFB will be administered at study enrollment (baseline) and monthly by phone to record opioid misuse over the 6-month RCT. TLFB offers more granularity for days-per-month of opioid misuse than a quantitative urinalysis test, but is subject to misreporting. Days of opioid misuse will be collected monthly in the 6-month study.

#### Tertiary outcome variables.

We will also conduct survival analyses to determine if time to opioid lapse (from quantitative urinalysis results) or time to treatment discontinuation (from clinical records) differs between study arms. Finally, participants in the MOUD+OptiMAT arm will participate in qualitative interviews at month 2 about their OptiMAT usage including preferred features. We will evaluate if OptiMAT usage patterns (from quantitative smartphone data collected or the qualitative interviews) predicts opioid misuse in the MOUD+OptiMAT arm, as described below.

### Participant timeline {13}

Participants will participate in the study for 6 months. Table 1 summarizes the method and timing of data collection.

**Table:** 

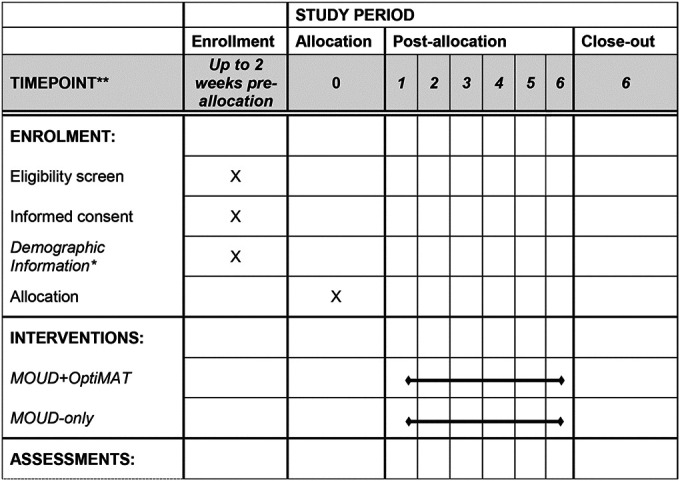 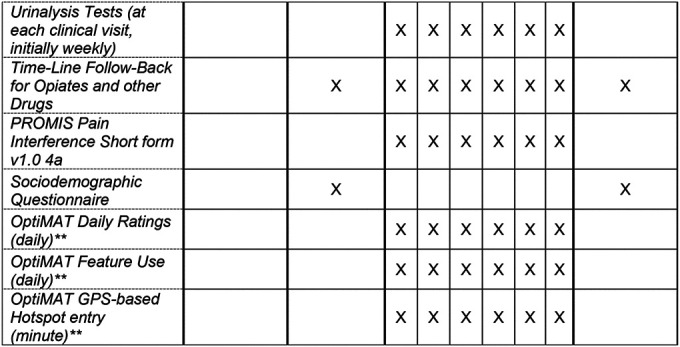

* Includes BIRC Demographic form, clinician report of comorbid alcohol or marijuana use, Alcohol Use Disorders Identification Test, Cannabis Use Disorders Identification Test, PROMIS Pain Interference Short form v1.0 4a.

** MOUD+OptiMAT arm only.

### Sample size {14}

As brief interventions, smartphone apps have large effect sizes (Cohen’s δ) for aiding weight loss (δ≥ 0.60), but weaker effects for aiding smoking cessation (δ≈ 0.16–0.28) and alcohol reduction (δ≈ 0.23–0.40).(9,24–26) Our pilot OnTrack study suggested similar effect sizes for reducing alcohol and marijuana use (both δ≈0.26) and greater effect sizes for reducing risky sexual behavior (δ≈ 0.70).(21) A clinical trial using the CHESS-A smartphone app for reducing opioid misuse estimates an effect size for reducing opioid use of δ= 0.35, which is comparable to reducing use of other drugs of abuse. Assuming Cohen’s δ=0.35, 1:1 randomization into the two study arms, a Type I error rate of 0.05 for comparisons of the mean difference between the two groups using independent t-test, a statistical power of 0.80 could be achieved with accrual of N=260 participants. Assuming a 20% drop-out rate and stratification of random study arm assignment across 12 variable levels (up to 3 recruitment sites × 2 chronic pain levels × 2 levels of comorbid use of marijuana or alcohol), this accrual goal could be reached with enrollment of 336 participants.

At this time, there is insufficient effect size and variance data to conduct an informed power analysis for using GEMA to support location-based intervention. We will report post-hoc effect sizes from data acquired from this project.

### Recruitment {15}

We have obtained a partial waiver of HIPAA authorization so that clinical staff may identify patients meeting eligibility criteria and introduce those individuals to an on-site research coordinator. Recruitment and enrollment will be conducted by research staff rather than clinical staff to avoid coercion. The research coordinator will provide patients with IRB-approved advertisements describing the study. The research coordinator will also schedule an intake visit for interested patients, where patients will undergo the informed consent process (including full HIPAA authorization) in a private and confidential setting. We will also obtain a partial HIPAA waiver to allow undecided participants to provide us with contact information (i.e., name and phone number) so that we may recontact them later. Patients who provide written informed consent to participate in the study will be randomized into one of two intervention arms (see Allocation below).

## Assignment of interventions: allocation

### Sequence generation {16a}

Randomization of participants into study arms (MAT+OptiMAT or MAT-only) will be stratified by two variables: chronic pain and comorbid use of alcohol and/or marijuana, to address the possibility that these variables may influence treatment outcomes. Chronic pain is prevalent in this population, with 40% of our pilot participants reporting that pain has moderate or severe interference with daily activities (PROMIS Pain Interference Short form v4a). Patients receiving MOUD who have comorbid alcohol or marijuana use are placed on a slower progression from Phase I to Phase II to reduce potential misuse of take-home Suboxone. Chronic pain will be defined by pain intensity (none/low vs. moderate/severe on PROMIS Pain Interference Short form v4a) and comorbid alcohol or marijuana use will be defined as yes vs. no, as reported by clinical staff. We will document severity of alcohol or marijuana misuse with the Alcohol Use Disorders Identification Test (AUDIT) and Cannabis Use Disorders Identification Test (CUDIT), but will rely on clinical staff report for randomization. Randomization within each of the four strata (low pain +no comorbidity; low pain +comorbidity; high pain +no comorbidity; high pain +comorbidity) will be determined at study initiation by 1:1 allocation ratio using R software package blockrand which can be used to produce randomization lists and cards.

### Concealment mechanism {16b}

The randomization order for each site will be written on notecards; sealed in opaque, consecutively numbered envelopes, and placed sequentially in a lockbox at each site.

### Implementation {16c}

Our biostatistician (XH) will create the randomization order for each site. The principal Investigator (GAJ) will transcribe the randomization orders into sealed envelopes as described above. Study staff will allocate participants to intervention arms during the Intake visit.

## Assignment of interventions: Blinding

### Who will be blinded {17a}

Blinding of patients and clinical providers is not possible for this RCT, given that participants will be aware of their study arm assignment and may volunteer this knowledge to clinical providers or other patients during group or individual therapy sessions. We propose a single blind in which the clinical (laboratory) staff conducting quantitative urinalysis for opioid misuse are unaware of participant group membership. Additionally, the biostatistician (XH) responsible for analyzing primary and secondary outcome measures will be blind to group labels.

### Procedure for unblinding if needed {17b}

N/A. Only clinical (laboratory) staff and the biostatistician are blinded. We foresee no circumstances in which unblinding of these staff and investigator are permissible.

## Data collection and management

### Plans for assessment and collection of outcomes {18a}

The primary outcome variable, quantitative urinalysis drug testing, will be acquired and analysed by clinical staff per standard clinical procedure (see Primary Outcome, above).

The secondary outcome variable Time to Opioid Lapse will be derived from quantitative urinalysis drug test results above. The secondary outcome Time to Treatment Discontinuation will be calculated from clinical staff as the first of three consecutively missed clinical appointments. The secondary outcome self-reported days of opioid misuse will be calculated from the monthly Time-Line Follow-Back calendar assessments, collected via phone by research staff.

### Plans to promote participant retention and complete follow-up {18b}

Study staff will contact participants by text and phone throughout their 6-month participation in this study. Clinical sites include Peer Specialists (patients in long-term recovery from opioid use disorder) who serve as liaisons between the clinics and patients to provide support and guidance while encouraging recovery. Peer specialists also provide appointment reminders and encourage accountability in attending clinical visits.

### Data management {19}

These data will be stored in patients’ electronic medical records (EMRs). All other surveys and instruments will be administered by research staff using REDCap (Research Electronic Data Capture) database, a secure web-based database for collecting and storing survey-based research data.(27) REDCap includes logic to detect incorrect data entry such as range check for data values. Research staff will manually enter urinalysis results from EMRs into REDCap with double data entry. For privacy and confidentiality, the REDCap database will store information by coded study ID without PHI linkages.

### Confidentiality {27}

The Principal Investigator will carefully monitor study procedures to protect the safety of research subjects, the quality of the data and the integrity of the study.

#### Protection Against Loss of Confidentiality.

OptiMAT assessments: The OptiMAT app will serve as a portal to a secure database storing all data entered by the participants. Any data entered into the OptiMAT app will be uploaded by secure HTTPS connection to a HIPAA-compliant, HITRUST certified secure database managed by Enqbator. If the smartphone is on a secure private wireless network (like their home network or their provider’s cellular network), then the entered data will automatically upload to the server. If the smartphone is not on a secure private wireless network (e.g. a public network, like at a coffee shop), then the data will be stored on the phone locally until the smartphone is connected to a private wireless network or cellular network, at which point it will be automatically uploaded to server. This transfer process will happen automatically. Second, the participant will always be able to delete the OptiMAT app to prevent loss of confidentiality without losing data collected on the server. Third, study staff will require participants to enable passcode protected lockscreens on their phones, and warn them about potential loss of privacy if they disable the passcode protected lockscreens. Finally, OptiMAT will store data using only study codes (no PHI) to further protect privacy and confidentiality.

Enqbator server and data access: Enqbator retains no rights to the study data uploaded to its server. At the conclusion of this study, the PI will download all study data from the Enqbator’s server, and Enqbator will delete the data on the server. All data on Enqbator server will be stored using study ID. The only potentially identifying information on the server is GPS location, which may be used to infer a home address or work address. Enqbator and UAMS have entered into a Master Services legal agreement specifying that Enqbator will “keep the Disclosing Party’s (UAMS) Confidential Information confidential, and will not use or disclose such information to any third party for any purpose except (i) as expressly authorized by the Disclosing Party in writing, or (ii) as needed to fulfill the Receiving Party’s obligations under this Agreement.”

In-person assessments: To protect the identity of research volunteers, identifying contact information would be stored in a file (electronic) or in a locked cabinet (paper consent forms) separate from experimental data forms. All experimental data would be de-identified using an alphanumeric study code rather than a name or other identifying information. All data collection forms would be stored in a locked cabinet in the in a key card-protected room in the BIRC. Computer data records would be stored in password-protected network drives accessible only by study personnel. Data would be stripped of all identifiers, including the stripping of facial features from the anatomical MRI data. As a further measure to protect against the compelled disclosure of personally identifiable information, the investigative team has a NIDA-supported Certificate of Confidentiality. All personnel involved in the conduct of the proposed research would comply with the applicable Federal regulation for the protection of human subjects or, if no such federal regulation is otherwise applicable, they would comply with 45 CFR Part 46.

UAMS IRB policy considers all study personnel as mandated reporters, requiring that study personnel report incidents of child abuse and neglect to the Arkansas Department of Human Services. Study personnel must also report intentions to hurt others to local authorities. Subjects will be informed of this during the consent process, and that incidents of child abuse/neglect or intentions to harm others are not covered by the Certificate of Confidentiality.

### Plans for collection, laboratory evaluation and storage of biological specimens for genetic or molecular analysis in this trial/future use {33}

Not applicable: no biological specimens will be collected for research during this study.

## Statistical methods

### Statistical methods for primary and secondary outcomes {20a}

#### Analyses of primary and secondary outcomes.

The adequacy of randomization will be assessed by comparing baseline variables with known prognostic importance between the MOUD+OptiMAT and MOUD-only study arms. These baseline variables include demographic variables (age, sex, etc.) and clinical variables (Suboxone dose at initiation, initiating MOUD in a residential setting prior to initiating outpatient MOUD, etc.). An imbalance between the study arms is suspected if the proportion of variables with significant differences (p<0.05) exceeds 5%. We will then run the statistical models below with and without those variables identified as being associated with imbalance between the groups included and compare the results. If the results from both approaches are similar, we will use the models without those variables included. Otherwise, we will adjust those variables in the models.

We will next assess missing data. If the percentage of missing data is large, multiple imputation will be performed if the missing pattern is missing at random. If the pattern is not missing at random (NMAR), other methods like Bayesian models will be used. Distributions of outcome variables will also be assessed using appropriate tests and visual graphs. Linearity between continuous covariates and outcomes will be checked and other functional forms of the covariates will be used if the linear relationships are not shown.

General linear or generalized linear models (GLMs) will be used to examine associations between study arms and primary outcome (percent positive urinalysis tests) at the 6-month endpoint depending on whether the outcome is normally distributed or not. The model will include the indicator variable for study arm (MAT+OptiMAT vs. MAT-only) and the three stratification variables used for randomization (Site, Chronic Pain, and comorbid Alcohol or Marijuana Use) only first. Then the model will be run again with any additional covariates that were identified as being unbalanced between the study arms, and results will be compared.

For the secondary outcome of days-per-month of opioid misuse over the course of the 6-month study period, we will use generalized linear mixed models (GLMMs) to account for multiple records within each participant with Poisson or negative binomial distribution specified (as the outcome is probably skewed). GLMM will allow us to examine if overall days-per-month of opioid misuse varies between study arms and if this difference varies by study month. The models will include the indicator variable for MAT+OptiMAT vs. MAT-only, time with values 1 to 6 representing each of the six outcome variables measured monthly, time by study arm indicator interaction, strata variables and baseline days of misuse without and with additional covariates identified above added. The results from both models will then be compared.

If the percentage of missing data is deemed large and missing is not at random (MNAR) or if the planned sample size is not met, we will then use Bayesian methods for the analyses as Bayesian methods are more robust for small samples and can handle MNAR data by modeling the missing values. Posterior mean parameter ratios and posterior probabilities of the mean parameter ratios in the log scale being positive will be calculated. Bayesian models will include the models for outcomes and the models for indicator variables for missing values. The same variables as described in the previous paragraphs will be included. For the secondary outcome of days-per-month of opioid misuse, models will be fit separately for days of opioid misuse at each month with corresponding models fitted for the missing outcomes. Non-informative priors will be specified for all parameters. Convergence diagnostics tests such as Geweke, Raftery-Lewis, Heidelberger-Welch, Effective Sample Size, and posterior autocorrelations will be used to evaluate Markov chain convergence. Visual trace plots will also be used to aid evaluation of Markov chain convergence.

Survival analyses will be performed using the Kaplan-Meier method to compare the study arms on secondary outcomes (1) time to opioid lapse and (2) time to treatment discontinuation. Loss to follow-up will not be coded as relapse and will thus be censored in the Kaplan-Meier survival analyses for opioid lapse, but will be coded as treatment discontinuation. As with the analysis above, stratification variables (Site, Chronic Pain, and Comorbid Alcohol or Marijuana Use) will be included in the survival analyses only first. Then covariates identified above will be added and results will be compared. All analyses will be performed using SAS 9.4.

### Interim analyses {21b}

We have established a Data Safety Monitoring Board (DSMB) to monitor treatment outcomes (see {23}). The study biostatistician (XH) will prepare interim analyses for the DSMB while blinded to group membership, which the study PI (GAJ) will present unblinded to the DSMB. The DSMB will make recommendations concerning study stoppage, as warranted by data and ethical considerations, to the UAMS IRB. DSMB recommendations and PI’s response will be shared with NIH via annual progress reports, and reported to ClinicalTrials.gov.

### Methods for additional analyses (e.g. subgroup analyses) {20b}

#### Subanalyses of OptiMAT usage patterns predicting outcome.

Subanalyses will evaluate if usage of specific OptiMAT features predict treatment outcomes. Missing data and data normality will be assessed as described above, with methods adjusted accordingly. First, GLMM will evaluate if concurrent opioid misuse can be predicted from daily ratings of opioid craving, opioid withdrawal, stress, anger, sadness, alcohol use, and/or marijuana use. GLMM will also evaluate if these variables can predict subsequent opioid misuse (within the next week). Second, GLMM analysis of GPS data will evaluate if opioid misuse can be acutely predicted by entering hotspots, duration of time spent in hotspots, or craving ratings while in hotspots.

Third, qualitative interviews conducted at the 2-month endpoint will evaluate which OptiMAT features (e.g. the Tip Bank, Graphs of daily ratings, sobriety resources) participants report as most beneficial, and which of these features most reliably predict 6-month outcome. We will use established procedures to enhance the trustworthiness of our analysis, including triangulation of data generated from multiple methods and participants, team de-briefings, prolonged engagement with study site, and development of an audit trail. Interviews will be digitally recorded, de-identified, transcribed, and uploaded to MAXQDA qualitative data analytic software (http://maxqda.com/). We will conduct conventional content analyses to create preliminary *a priori* and emergent themes within which to sort participant quotes.(28) Two coders will code interviews to enhance rigor in analysis. First, we will develop a preliminary codebook consisting of top and sub-level codes after reading 2–3 transcripts.(29) Then, we will independently code 10% of transcripts and compare results; through discussion, we will refine the codebook. Next, we will independently code a different 10% of transcripts and compare results with interrater reliability. If interrater reliability is acceptable, with consensus, we will divide and independently code remaining transcripts to determine final themes. To further develop the thematic templates once interviewing has begun, coding will occur in an iterative fashion, with the templates being expanded and refined. We will also employ a quick and comprehensive qualitative analysis strategy, termed the rigorous and accelerated data reduction (RADaR) technique,(30) which involves using tables and spreadsheets from general-purpose word-processing software to develop all-inclusive data tables. These tables undergo several revisions, called “data reduction,” which produces more concise data tables. The RADaR technique: converts raw, textual data in to a more manageable and user-friendly format; is rigorous because of the systematic analysis that occurs at each step; and is accelerated because the time required to review and reduce each phase of the data table becomes shorter as the user produces more condensed and concise presentations of the textual data. Ultimately, we will produce “final” templates that will be applied to all of the interview data using MAXQDA qualitative coding software.(31) Once the templates have been applied to the transcripts, we will perform interpretive analyses to connect and contextualize the themes/recommendations and map out relationships across themes/recommendations.

### Methods in analysis to handle protocol non-adherence and any statistical methods to handle missing data {20c}

We will assess missing data as described in {20}. If the percentage of missing data is large, multiple imputation will be performed if the missing pattern is missing at random. If the pattern is not missing at random (NMAR), other methods like Bayesian models will be used. Distributions of outcome variables will also be assessed using appropriate tests and visual graphs. Linearity between continuous covariates and outcomes will be checked and other functional forms of the covariates will be used if the linear relationships are not shown.

### Plans to give access to the full protocol, participant level-data and statistical code {31c}

To promote study transparency and open science, published findings will include links to deidentified BIDS-compliant datasets and statistical code used for analyses.

## Oversight and monitoring

### Composition of the coordinating centre and trial steering committee {5d}

The Principal Investigator Dr. James will meet as needed (at least weekly) with study staff to review study progress. Dr. James will work with study staff to troubleshoot and resolve issues relating to study recruitment and day-to-day conduct. Dr. James meets weekly with Drs. Thompson and Bollinger (co-creators of the OptiMAT smartphone app) and monthly with all study co-investigators and consultant to discuss study progress.

### Composition of the data monitoring committee, its role and reporting structure {21a}

The DSMB will include representation from the UAMS Institutional Review Board (IRB) and UAMS departments of Psychiatry, Biostatistics, and Radiology. The DSMB will meet annually to review study-related AEs and SAEs. The study Biostatistician (XH) will prepare interim analyses for the DSMB while blinded to group membership, which Dr. James will present unblinded to the DSMB. The DSMB will make recommendations concerning study stoppage, as warranted by data and ethical considerations, to the UAMS IRB. DSMB recommendations and PI’s response will be shared with NIH via annual progress reports, and reported to ClinicalTrials.gov. The DSMB and IRB are independent from the sponsor and competing interests.

### Adverse event reporting and harms {22}

Adverse events (AE), serious adverse events (SAE), and unanticipated problems involving risks to subjects or others (UPIRSO) will be reported per UAMS IRB policy. Briefly, staff members will complete an initial AE report upon learning that an AE has occurred. The PI Dr. James will review individual AE reports briefly, and discuss these reports at a weekly meeting with co-investigators Drs. Thompson and Bollinger. Drs. James, Thompson, and Bollinger will determine if an AE meets the criteria for a SAE or UPIRSO. UAMS IRB policy states that an AE should be considered an UPIRSO if it was unanticipated, related to the research, and involves some increased risk to the subject. The PI will report AEs annually to the IRB at continuing review and to NIDA during the annual progress report. The PI will report SAEs and UPIRSOs to the IRB and NIDA PO within 10 days of learning of the event. UAMS IRB and/or NIDA may request modification of RCT procedures in response to the event. The PI and study team will follow all AEs to satisfactory resolution by the UAMS IRB. This may include withdrawing a subject if the investigative team determines that doing so is the best decision to protect the participant’s safety. Participants withdrawn from the RCT due to an SAE will have appropriate follow-up medical monitoring, which will be reported to the UAMS IRB and NIDA.

### Frequency and plans for auditing trial conduct {23}

Per UAMS policy, study data will be available to the UAMS Office of Research Compliance (ORC) at all times for review. ORC may conduct audits of study procedures. Findings from these audits will be reported to the UAMS IRB for review of study safety and protocol deviations. The IRB will communicate audit-related decisions to the PI in a timely manner. In the IRB takes an action that impacts the day-to-day operations of the trial (e.g., suspends recruitment, halts the RCT), the PI will report those actions to the NIDA program officer within 3 business days of receipt, consistent with NIDA policy.

### Plans for communicating important protocol amendments to relevant parties (e.g. trial participants, ethical committees) {25}

Per UAMS policy, the IRB will review all proposed protocol modifications. IRB reserves the right to deny unjustified protocol modifications. If the IRB determines that a protocol modification or DSMB committee report impacts the study risk-benefit ratio, the IRB may require that the modification or DSMB report be provided in writing to current and/or former study participants. Protocol modifications will be updated as they occur in ClinicalTrials.gov and reported annually to NIDA via continuing review.

### Dissemination plans {31a}

Trial results will primarily be disseminated through peer-reviewed publications. Trial results may also be communicated via media interviews, news releases, or conference proceedings.

## Discussion

We have described our planned protocol for a randomized controlled trial evaluating adjunctive use of smartphone app OptiMAT to improve 6-month outcomes for patients receiving MAT for OUD. We believe that OptiMAT’s use of daily monitoring, personalized feedback, and GPS-driven just-in-time intervention will reduce opioid misuse during this 6-month trial.

Our trial design includes several best practices for RCT methodology, including: stratification of study arm assignment by key study variables suspected to influence outcome (comorbid pain interference and comorbid use of alcohol or marijuana); reliance on the Treatment Effectiveness Score for primary outcome of negative urinalysis test; data safety monitoring; and inclusion of secondary outcomes for participants who discontinue MOUD treatment. We acknowledge that study confounds may still arise despite these best practices, and provide an adaptable statistical plan that can be tailored to the specific data properties of the resulting dataset (e.g. adjusting for non-normal distributions and patterns of missing data).

We additionally provide subanalyses for evaluating the OptiMAT features and usage patterns that best predict 6-month outcomes. These subanalyses seek to identify the mechanisms by which app-based interventions promote positive outcomes, and thus identify the critical components for future app interventions.

## Trial status

This trial is operated under UAMS IRB protocol #274084, v4, approval date July 11, 2022. Recruitment is anticipated to begin February 2022 and conclude September 2027. Trial status will be continuously updated through its registration at ClinicalTrials.gov (Identifier: NCT05336188, https://clinicaltrials.gov/ct2/show/NCT05336188).

## Data Availability

To promote study transparency and open science, published findings will include links to publicly available, deidentified BIDS-compliant datasets and statistical code used for analyses.

## Abbreviations

AE: adverse events
DSMB: data safety monitoring board
GEMA: geographical ecological momentary assessment
GLM: generalized linear models
GLMM: generalized linear mixed models
EMR: electronic medical record
MAT: medication assisted treatment
MATRIARC: Medication Assisted Treatment Recovery Initiative for Arkansas Rural Communities
MNAR: missing not at random
MOUD: medication assisted treatment for opioid use disorder
OptiMAT: “Optimizing Medication Assisted Treatment” smartphone app
OUD: opioid use disorder
RCT: Randomized controlled trial
REDCap: Research Electronic Data Capture
SAE: serious adverse events
TLBF: Time-Line Follow-Back calendar of drug use
TES: Treatment Effectiveness Score
UAMS: University of Arkansas for Medical Sciences
UAMS CAST: UAMS Center for Addiction Services and Treatment
UAMS BIRC: UAMS Brain Imaging Research Center
UAMS PRI: UAMS Psychiatric Research Institute
